# Pure Ankle Dislocation: A Case Report and a Narrative Literature Review

**DOI:** 10.7759/cureus.43071

**Published:** 2023-08-07

**Authors:** Constantinos Chaniotakis, Alexandros Tsioupros, Kosmas Samartzidis, Kalliopi Alpantaki, Ioannis M Stavrakakis

**Affiliations:** 1 Department of Orthopedics and Traumatology, Venizeleio General Hospital, Heraklion, GRC

**Keywords:** anterior talo-fibular ligament, calcaneofibular ligament, deltoid ligament, tibiotalar dislocation, posteromedial ankle dislocation, pure ankle dislocation

## Abstract

Pure dislocation of the ankle is an extremely rare injury accounting for only 0.065% of all ankle injuries and 0.46% of all ankle dislocations. The mechanism of the injury generally consists of high-energy trauma which is associated with a combination of plantar flexion and inversion or eversion of the foot.

We present a case of a 22-year-old male patient who sustained a closed pure ankle dislocation after a fall from a small height. He was treated conservatively with closed reduction and circumferential cast immobilization for six weeks, followed by a functional rehabilitation program.

The patient presented to the emergency department with an acutely painful and deformed right ankle after falling from a height of 1 m (stairs). Radiographs showed a posteromedial ankle dislocation without fracture. Urgent closed reduction of the dislocation was performed and a posterior below-knee back slab was applied to immobilize the ankle. Dorsalis pedis and posterior tibial arteries were intact. Check X-rays confirmed proper reduction of the ankle joint. Post reduction computed tomography (CT) scan did not show any associated fractures. Magnetic resonance imaging (MRI) revealed a multiligamentous ankle injury and a small osteochondral lesion of the anteromedial talar dome. The back slab was changed to a below-knee circular cast two weeks later, as soon as the soft tissue swelling subsided. The cast was removed at the six-week follow-up and physiotherapy was initiated in order to gain functional rehabilitation and improve the range of motion. At the final follow-up (12 months), the ankle range of motion (ROM) was the same as the pre-injury status and the patient was able to return to his work.

Pure ankle dislocation is a rare injury. A satisfactory outcome can be expected, provided that the appropriate conservative treatment followed by a strict rehabilitation protocol is applied.

## Introduction

Ligamentous injuries of the ankle joint as well as malleolar fractures are common, but ankle dislocation without associated fracture, also known as pure ankle dislocation, is an extremely rare injury [1]. Pure ankle dislocation is not well understood due to its rarity. In the literature, only 154 cases were reported up to 2017 [2,3]. In a systematic review of Wight et al., the incidence of pure ankle dislocation accounted for 0.065% of all ankle injuries and 0.46% of all presentations with an ankle dislocation [4]. Typically, these injuries are the result of high-energy trauma, such as motor vehicle accidents, falls from height and sports traumas, where the failure of the ligamentous stabilizers predominates [5-7]. Although the ankle joint has intrinsic stability (the mechanical efficiency of the ankle mortise, ankle ligaments and the strong capsule), anatomical defects such as hypoplasia of medial malleolus, atrophy of the peroneal muscles, ligamentous laxity and recurrent ankle sprain predispose the ankle joint to dislocation [8,9].

In 1965, Fahey and Murphy classified tibiotalar dislocations into five types based on the direction of the dislocation (anterior, posterior, medial, lateral, superior or combination) [10,11]. The most common type of pure ankle dislocation is posteromedial [12]. For example, a fall from a height can usually result in a posteromedial dislocation due to axial loading, plantarflexion, and inversion of the ankle [13]. Plain X-rays (anteroposterior and lateral views) should be obtained before and after the reduction maneuver. Mortise view is obtained after the reduction for the distal tibio-fibular syndesmosis evaluation. Computed Tomography (CT) is necessary to rule out the presence of malleoli fractures and it should be obtained after the reduction. The clinical examination includes evaluation of the skin (closed or open injury, bony compression of the skin and swelling), and neurovascular status of the foot [14].

Closed reduction and cast immobilization of these injuries, under sedation, is important to cease pressure on the neurovascular structures and on the skin [15]. Although the gold standard treatment (conservative or surgical) for these rare injuries has not yet been established, conservative treatment seems to be the most optimal way of managing closed injuries [16,17]. Prognosis is good with the appropriate treatment and rehabilitation protocol in most cases [5].

We present a case of a 22-year-old male patient who sustained a closed pure ankle dislocation after a fall from a small height. He was treated conservatively with closed reduction and circumferential cast immobilization for six weeks, followed by a functional rehabilitation program, with a satisfactory outcome. A review of the literature is also presented.

## Case presentation

**Figure 1:**
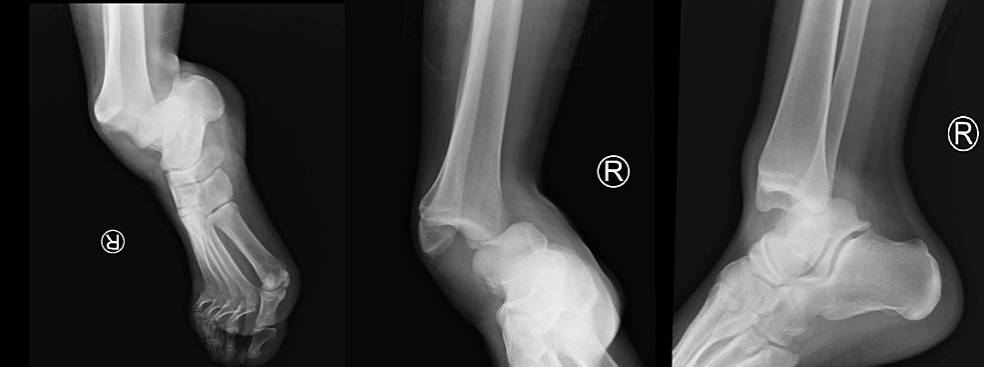
Posteromedial pure ankle dislocation. Anteroposterior and lateral radiographs.

A 22-year-old male patient presented to the emergency department in May 2022, after a fall from a height of 1 m (stairs). He complained of right ankle pain and inability to bear weight immediately after the injury. His past medical history was unremarkable. He described the injury as an axial loading, ankle plantarflexion, suspension of the lateral ray and foot supination. There was no open injury, but swelling was present. The foot was displaced in a posteromedial direction. The neurological examination could not be performed because of severe pain. Plain anterior-posterior and lateral radiographs of the ankle demonstrated a posteromedial dislocation of the ankle, without any fracture (Figure 1). Urgent closed reduction was performed under sedation using gentle traction followed by lateral compression and external rotation of the talus and dorsiflexion of the foot. On examination, which was performed post reduction maneuver, dorsalis pedis and posterior tibial arteries were intact.

**Figure 2:**
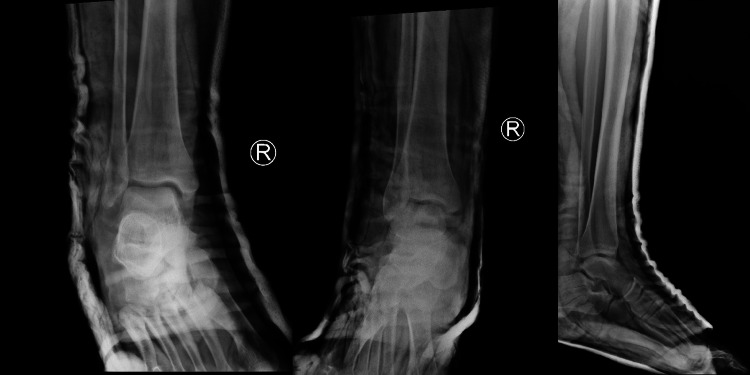
Post reduction radiographs of ankle.

**Figure 3:**
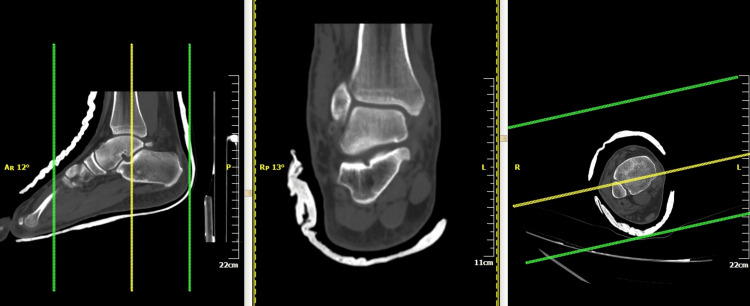
Post reduction CT scan of ankle was used to exclude associated fractures.

**Figure 4:**
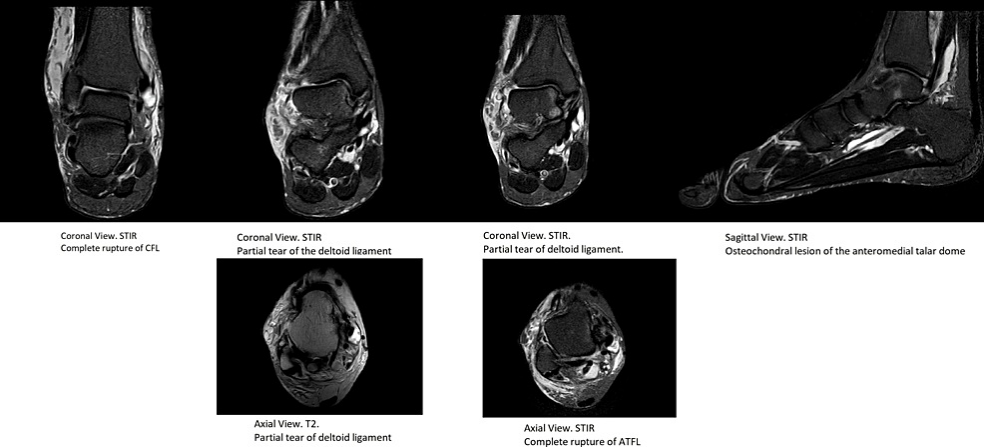
Post reduction MRI revealed a multiligamentous ankle injury without associated fractures. The osteochondral lesion of the anteromedial talar dome. ATFL: anterior talo-fibular ligament (ATFL); CFL: calcaneofibular ligament

Post reduction stress test of the ankle revealed an anterolateral instability. A posterior below-knee back slab was applied to immobilize the ankle. The use of circular cast was not applied due to the swelling of ankle. Post manipulation X-rays confirmed proper reduction of the ankle joint, without any fracture or widening of the tibio-fibular syndesmosis (Figure 2). Post reduction CT scan did not show any associated fractures (Figure 3). MRI revealed a complete rupture of the anterior talo-fibular ligament (ATFL) and calcaneofibular ligament (CFL) laterally, partial tear of the deltoid ligament medially, and small osteochondral lesion of the anteromedial talar dome (Figure 4).

**Figure 5:**
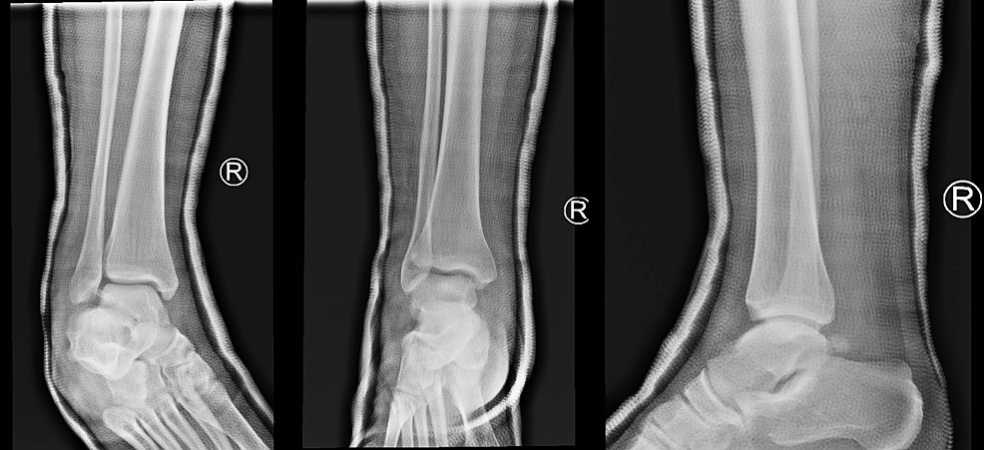
Plain X-rays at two-week follow-up. The back slab was changed for below-knee circular cast.

**Figure 6:**
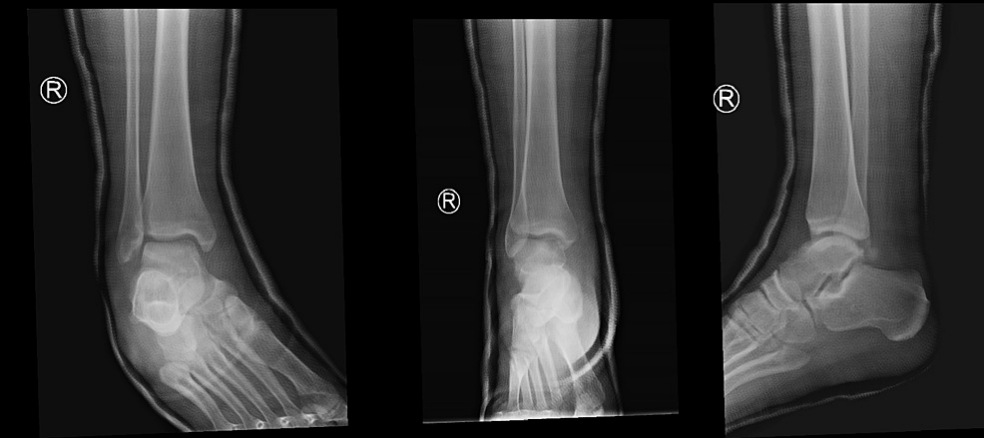
Plain X-rays at six-week follow-up.

Elevation of the right lower extremity, ice therapy and oral analgesics were used in the early post reduction period. We administrated enoxaparin sodium for six weeks (inj. sol. 4,000 IU anti-Xa, 0.4ml, s:1x1). After two days of hospitalization, the patient was discharged without any complications. The back slab was changed for below-knee circular cast two weeks later, as soon as the soft tissue swelling subsided (Figure 5). The cast was removed at the six-week follow-up (Figure 6). Recommended physiotherapy was started in order to gain functional rehabilitation. The type of physiotherapy was ankle range of motion (ROM) exercises and peroneal muscle strengthening. At six weeks, the patient started full weight-bearing as tolerated. At the final follow-up (12 months), the ankle ROM was the same as the pre-injury status, without pain or any impingement in the clinical examination and he was able to return to his work. It was considered that the osteochondral lesion had healed because the patient did not report any symptoms (pain) and we did not use MRI to evaluate the osteochondral lesion healing.

## Discussion

In 1913, Peraire presented the first case of tibiotalar dislocation without an accompanying fracture of the ankle [18]. Pure ankle dislocation remains a rare injury and only small case series have been reported in the literature [3,19,20,21]. We present the incidence of several types of pure ankle dislocation according to case series studies found in the literature in Table 1.

**Table 1:** The incidence of several types of pure ankle dislocation.

Authors	Number of dislocations	Type of dislocation	Open/ Closed
Karampinas PK et al. [1]	1	Anterior-lateral	Closed
Bouziane W et al. [2]	1	Posteromedial	Closed
Gan TJ et al. [3]	8	Posteromedial	Six open, Two closed
Lima AGDB et al. [5]	1	Posterior	Closed
Rivera F et al. [6]	3	One posteromedial, One lateral, One posterior	Two open, One closed
Almotlaqem N et al. [7]	1	Posteromedial	Closed
Ri’os-Luna A et al [8]	2	One anteromedial One Posterior	One open, One closed
Fotiadis E et al [9]	1	Posterior	Closed
Luthfi APWY et al [10]	1	Posteromedial	Open
Wang YT et al. [12]	3	Posteromedial	Closed
Hatori M et al. [13]	1	Posteromedial	Open
Mubark I et al. [15]	1	Posterior	Closed
Kawai R et al [16]	1	Medial	Closed
Lui TH et al. [19]	6	Posteromedial	Open
Garbuio P et al [20]	9	Six medial and posteromedial	Seven open, Two closed
Moehring HD et al [21]	14	Posteromedial	Open
Elisé S et al [23]	16	Eight posteromedial, Four posterior, One anterior, One high variety, One of the Huguier type, One complex	Eight open, Eight closed
Wroble RR et al. [24]	9	Three medial, Two posteromedial, Two posterior, Two lateral	Four open, Five closed
Toohey JS et al. [25]	19	Four medial, Six lateral, Three posterior, One upward, Five indetermine	Six open Thirteen closed
Bhullar PS et al. [26]	1	Medial	Open
Dlimi F et al. [27]	1	Medial	Open
Tarantino U et al. [28]	1	Medial	Open
Uçar BY et al. [29]	5	Four medial, One posterior	Open

The posteromedial ankle dislocation represents the most common type of injury [12]. When a posterior force is applied to the tibia with forced dorsiflexion, an anterior dislocation usually occurs. Lateral and medial dislocations usually occur from forced eversion, inversion, or rotation of the ankle [1]. Fernades studied the mechanism of tibiotalar dislocation without fracture on cadaveric ankles. He also discussed that a tibiofibular syndesmotic disruption could occur when forced dorsiflexion of the foot is associated with external rotation and pronation of the ankle [22]. This mechanism of injury may cause a Maisonneuve type of fracture as well. In addition to this, Fernades suggested a pure ankle dislocation classification system based on the mechanism of injury (Table 2) [4].

**Table 2:** Classification system of pure ankle dislocation by Fernandes. [4]

Type	Direction	Mechanism
I	Medial, Posterior, Posteromedial	Axial loading, Plantarflexion, Internal rotation
II	Lateral	Axial loading, Plantarflexion, External rotation
III	Superior	Axial loading, Dorsiflexion, External rotation
IV	Anterior	Axial loading, Plantarflexion, Anterior force

Wight et al. noted the distribution of open and closed dislocations in a case series study of 154 pure ankle dislocations and 50% of all pure ankle dislocations were open. Infections (superficial or deep) occurred relatively commonly in open injuries, and they represented 8% of all complications. The most common complication of pure ankle dislocation was stiffness (18%) [4]. Post traumatic arthritis was the second most common complication with an incidence of 10%. In addition to this, Elisa et al. referred that osteoarthritis was reported in 25% of cases, notably after open dislocations [23]. Moehring et al. reported 14 open pure ankle dislocations with a long-term follow-up (mean 12 years) and concluded that despite the gross disruption of the ankle ligaments and associated soft tissue injury, overall outcomes are favorable [22]. Wroble et al. presented a retrospective review study of eight patients who suffered pure ankle dislocation. Calcifications and osteophytes were found in patients after a long follow-up period (range two to 24 years). In addition to this, authors estimated the overall incidence of vascular and neurological lesions at 10% [24]. Garbuio et al. reported nerve injury in five cases. In the dislocated foot, attention must be paid to vascular and neurological examination, because the foot’s viability is related to the vascular involvement [20].

**Figure 7:**
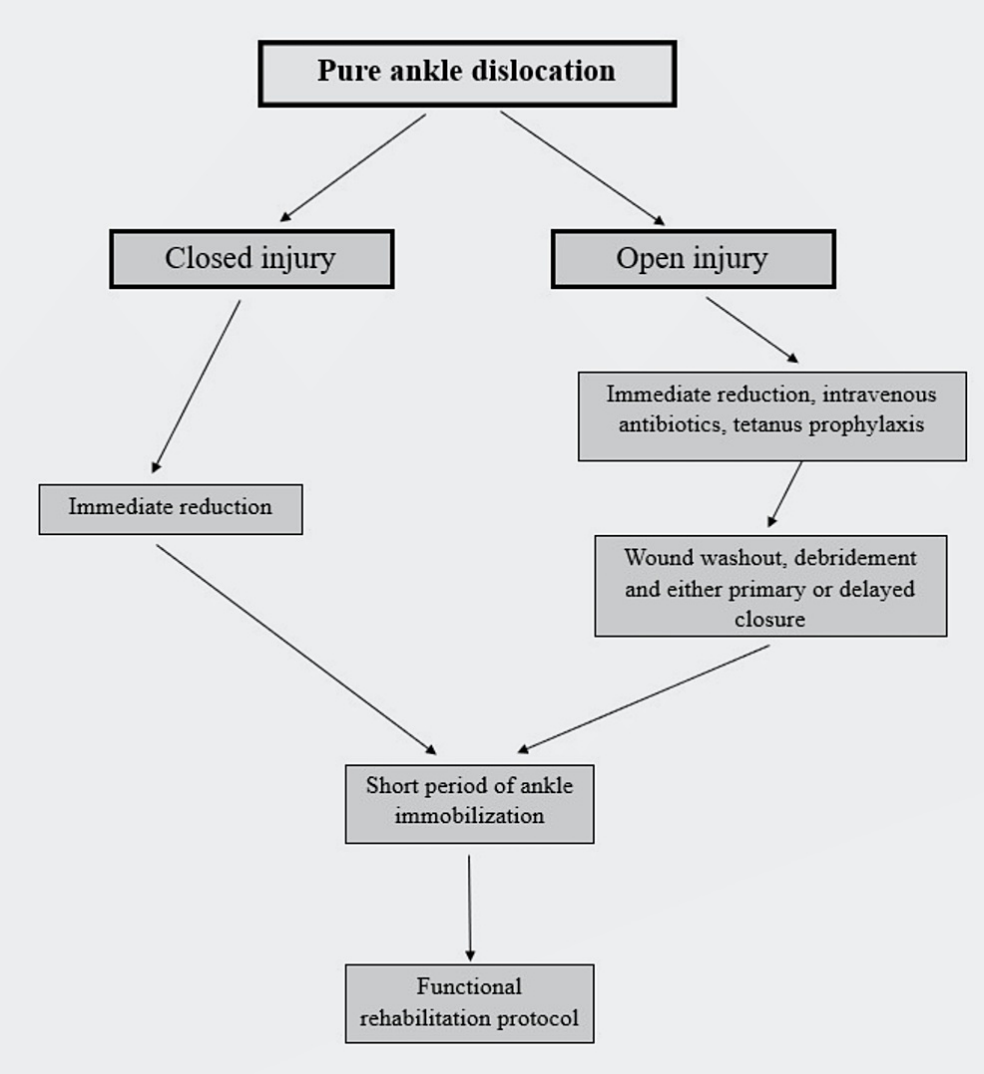
Recommendations for management of pure ankle dislocation (closed and open injuries) Wight et al. [4] This figure is an original creation by Constantinos Chaniotakis

The management of pure ankle dislocation appears to be straightforward, but there are controversies according to the type of injury (open or closed) [25]. Conservative treatment with early reduction followed by cast immobilization is recommended in closed injuries [16,17]. Our patient achieved full range of motion of the ankle, and he was able to return to his work at 12 months. However, the appropriate treatment (conservative or surgical) for patients with an open injury has not yet been established and various controversies have been reported in the literature about the role of primary ligamentous repair [26]. Some authors recommended the repair of the lateral ligaments during debridement. Dlimi et al. and Hatori et al. reported good function in their cases with open pure ankle dislocation that were treated with acute lateral ligamentous and capsular repair [13,27]. Rivera et al. presented good long-term results in patients treated with only repair of the ankle capsule [6]. In contrast to this approach, Tarantino et al. mentioned good function in a man who sustained an open pure ankle dislocation and was treated without ligamentous or capsular repair. This approach was based on the quality of ligaments and the authors noted that ligaments were shredded and contused. It was unnecessary to repair them [28]. Uçar et al. reported satisfactory outcomes without instability in patients who were treated without repair of the ligaments or the capsule [29]. Wight et al. presented management recommendations for pure ankle dislocations (Figure 7) [4]. 

## Conclusions

Pure ankle dislocations are extremely rare in adults. The contemporary literature reports only case reports and small case series. Early diagnosis and appropriate treatment are of paramount importance in order to achieve satisfactory outcomes. We agree with other authors that the proper conservative treatment provides good results in the majority of patients.

## References

[1] Karampinas PK, Stathopoulos IP, Vlamis J, Polyzois VD, Pneumatikos SG (2012). Conservative treatment of an anterior-lateral ankle dislocation without an associated fracture in a diabetic patient: a case report. Diabet Foot Ankle.

[2] Bouziane W, Amahtil M, Benhamou M (2022). Case report: a rare case of pure tibio-talar dislocation without malleolar clamp fracture in a young athlete: long-term functional results and recent reviews of the literature. Int J Surg Case Rep.

[3] Gan TJ, Li YX, Liu X, Zhang H (2022). Pure ankle dislocation without associated fracture: a series of cases and our clinical experience. Indian J Orthop.

[4] Wight L, Owen D, Goldbloom D, Knupp M (2017). Pure ankle dislocation: a systematic review of the literature and estimation of incidence. Injury.

[5] Lima AG, Petry Filho JC, Barbosa GM (2018). Tibiotalar dislocation without associated fractures: a case report. Sci J Foot Ankle.

[6] Rivera F, Bertone C, De Martino M, Pietrobono D, Ghisellini F (2001). Pure dislocation of the ankle: three case reports and literature review. Clin Orthop Relat Res.

[7] Almotlaqem N, Altammar A, Hassan A, Lari A (2023). Successful nonoperative treatment of a closed posteromedial ankle dislocation without associated fractures - a case report. Ann Med Surg (Lond).

[8] Ri’os-Luna A, Villanueva-Martinez M, Fahandezh-Saddi H, Pereiro J, Martin-Garcia A (2007). An isolated dislocation of the ankle: two cases and review of the literature.. Eur J Orthop Surg Traumatol.

[9] Fotiadis E, Kenanidis E, Hytas A, Lyrtzis C, Koimtzis M, Akritopoulou K, Samoladas E (2009). Surgical management of closed tibiotalar dislocation: a case report and 2-year follow-up. J Foot Ankle Surg.

[10] Luthfi AP, Maruanaya S, Dalitan IM, Wedhanto S (2023). Pure ligamentous ankle dislocation: a case report. Int J Surg Case Rep.

[11] Fahey JJ, Murphy JL (1965). Dislocations and fractures of the talus. Surg Clin North Am.

[12] Wang YT, Wu XT, Chen H (2013). Pure closed posteromedial dislocation of the tibiotalar joint without fracture. Orthop Surg.

[13] Hatori M, Kotajima S, Smith RA, Kokubun S (2006). Ankle dislocation without accompanying malleolar fracture. A case report. Ups J Med Sci.

[14] Frank AL, Charette RS, Groen K (2023). Ankle dislocation. StatPearls [Internet].

[15] Mubark I, Anwar S, Hayward K (2017). Closed posterior ankle dislocation without associated fractures: a case report. J Surg Case Rep.

[16] Kawai R, Kawashima I, Tsukada M, Tsukahara T, Aoshiba H (2020). Treatment of open ankle dislocation without associated fractures in a young athlete using external fixation and ligament repair with suture tape augmentation. BMC Musculoskelet Disord.

[17] Lamraski G, Clegg E (2010). Unusual upward closed tibiotalar dislocation without fracture: a case report. Foot Ankle Surg.

[18] Peraire A (1913). Luxation tibo-astragalienne avec issue à l’extérieur du péroné non fracturé à travers une boutonnière cutanée. Présentation de malade. Paris Chir.

[19] Lui TH, Chan KB (2012). Posteromedial ankle dislocation without malleolar fracture: a report of six cases. Injury.

[20] Garbuio P, Gerard F, Gagneux E (1995). [Pure dislocations of the tibio-talar joint. Apropos of 9 cases]. Rev Chir Orthop Reparatrice Appar Mot.

[21] Moehring HD, Tan RT, Marder RA, Lian G (1994). Ankle dislocation. J Orthop Trauma.

[22] Fernandes TJ (1976). The mechanism of talo-tibial dislocation without fracture. J Bone Joint Surg Br.

[23] Elisé S, Maynou C, Mestdagh H, Forgeois P, Labourdette P (1998). [Simple tibiotalar luxation. Apropos of 16 cases]. Acta Orthop Belg.

[24] Wroble RR, Nepola JV, Malvitz TA (1988). Ankle dislocation without fracture. Foot Ankle.

[25] Toohey JS, Worsing RA Jr (1989). A long-term follow-up study of tibiotalar dislocations without associated fractures. Clin Orthop Relat Res.

[26] Bhullar PS, Grant DR, Foreman M, Krueger CA (2014). Treatment of an open medial tibiotalar dislocation with no associated fracture. J Foot Ankle Surg.

[27] Dlimi F, Mahfoud M, Berrada MS, El Bardouni A, El Yaacoubi M (2011). Open medial ankle dislocation without associated fracture: a case report. Foot Ankle Surg.

[28] Tarantino U, Cannata G, Gasbarra E, Bondi L, Celi M, Iundusi R (2008). Open medial dislocation of the ankle without fracture. J Bone Joint Surg Br.

[29] Uçar BY, Necmioğlu S, Bulut M, Azboy I, Demirtaş A (2012). Open ankle dislocations without associated fracture. Foot Ankle Int.

